# Minimizing the risk of non-vertical, non-sexual HIV infection in children – beyond mother to child transmission

**DOI:** 10.7448/IAS.15.2.17377

**Published:** 2012-11-15

**Authors:** Mark F Cotton, Barend J Marais, Monique I Andersson, Brian Eley, Helena Rabie, Amy L Slogrove, Angela Dramowski, Hendrik Simon Schaaf, Shaheen Mehtar

**Affiliations:** 1 Division of Paediatric Infectious Diseases, Tygerberg Children's Hospital, Stellenbosch University, Cape Town, South Africa; 2 The Institute for Emerging Infectious Diseases and Biosecurity, the Clinical School, the Children's Hospital at Westmead, University of Sydney, NSW, Australia; 3 Division of Medical Virology, Department of Pathology, Stellenbosch University, Stellenbosch, South Africa; 4 Division of Infectious Diseases, Red Cross Children's Hospital, University of Cape Town, Cape Town, South Africa; 5 Department of Paediatrics and Child Health, Stellenbosch University, Stellenbosch, South Africa; 6 Division of Paediatric Infectious Diseases, Department of Paediatrics and Child Health, Stellenbosch University, Stellenbosch, South Africa; 7 Academic Unit for Infection Prevention and Control, Tygerberg Hospital, Stellenbosch University, Cape Town, South Africa

**Keywords:** HIV, non-vertical, non-sexual, transmission, premastication, infection control, injection safety, children

## Abstract

After witnessing an episode of poor injection safety in large numbers of children in a rural under-resourced hospital in Uganda, we briefly review our own experience and that of others in investigating HIV infection in children considered unlikely to be through commonly identified routes such as vertical transmission, sexual abuse or blood transfusion. In the majority of cases, parents are HIV uninfected. The cumulative experience suggests that the problem is real, but with relatively low frequency. Vertical transmission is the major route for HIV to children. However, factors such as poor injection safety, undocumented surrogate breast feeding, an HIV-infected adult feeding premasticated food to a weaning toddler, poor hygienic practice in the home and using unsterilised equipment for minor surgical or traditional procedures are of cumulative concern.

## Introduction

Mother to child transmssion (MTCT) of HIV occurs in utero, intra-partum or later via breast milk and is the most important route for HIV infection in children. Major strides have been made to reduce the rate of MTCT [1]. However, there are a number of other mechanisms of transmission. These include poor injection technique [2], blood transfusions [3], surrogate breastfeeding [4,5] and sexual abuse [6]. More recently, HIV transmission through premasticated food has been described [7,8]. Here, the mother can still be the source. The possibility of HIV transmission to children once MTCT has been excluded is insufficiently appreciated. In this article, we have adopted the term “non-vertical, non-sexual transmission” first introduced by Vaz *et al*. who investigated possible routes of HIV transmission to children with HIV-uninfected mothers and without a history of sexual abuse from Mozambique [9].

One of us (BJM) witnessed poor injection safety in a paediatric ward in a hospital while on a field trip in a malaria-endemic region of rural Uganda. The episode seemed representative of the situation in large parts of sub-Saharan Africa:

The ward was staffed by dedicated healthcare workers, but there were few nurses looking after a huge number of children in an overcrowded paediatric ward. Resources were scarce. Children were not provided with food. Since cots were limited, parents (and sometimes also younger siblings) mostly slept underneath their children's cots or children slept with their parents on the ground. Provision for hand washing was scarce. There was a single working tap outside the ward, but without soap. There was no alcohol hand disinfectant. Children, accompanied by their parents, were lined up to receive intravenous (IV) medication. All medications were withdrawn from multi-dose vials, mostly containing quinine, ampicillin or cloxacillin. Children had IV lines or locks for IV access and were injected with the same syringe (and needle in most instances). Needles were switched when moving from one vial to the next. The syringe was only discarded after all medications had been given. The nurse giving the IV medicine guided the needle into the IV hub with her ungloved finger, seemingly unaware of the grave infection risk.

This incident prompted us to review our own and others’ experience of possible non-vertical, non-sexual HIV in children, summarized in Table 1.

**Table 1:** Selected case series and surveys of children likely to have acquired HIV infection through non-vertical, non-sexual routes

Country and study	Number (%)	Study design	Comments
South Africa, Van Kooten Niekerk *et al*. [12]	8/274 (2.9%) HIV-infected children	Retrospective review of HIV-infected children at Tygerberg Children's Hospital, Western Cape	Maternal HIV identified through knowledge of mother's status
Uganda HIV/AIDS Sero-behavioural Survey 2004–2005 [18]	7/55 HIV-infected children	Cross-sectional national population survey	Numbers derived from statement that 0.1% of HIV-negative mothers had HIV-infected children (7271 mothers)
South Africa, Shisana *et al*. [5]	7/477 (1.46%) HIV-infected children	Cross-sectional prevalence survey of all HIV-infected children 2–9 years of age in Free State Province	Surrogate breast feeding or receiving expressed breast milk from a “milk room” in a hospital were the strongest risk factors
South Africa, Hiemstra *et al*. [13]	14 HIV-infected children	Case series. Two cases from beyond the Western Cape (Eastern Cape and Kwazulu-Natal)	Nosocomial origin implied for 13: 10 received expressed breast milk in hospital. Sexual abuse excluded through careful history, social worker interviews and physical examination. Blood products excluded through tracing and retesting of donors. (Includes 8 cases from Van Kooten Niekerk et al. [12])
Uganda, Biraro *et al*. [17]	1/26 (3.8%) HIV-infected children (non-vertical route possible for two additional children)	Cross-sectional surveillance data of children≤12 years of age in rural south-western Uganda	HIV-exposure by antibody determination. Early deaths of mothers were presumed HIV-related. The population attributable fraction not due to MTCT was between 6 and 10%.
Swaziland and Kenya, Okinyi *et al*. [19]	11/50 (22%) HIV-infected children. Eight HIV-infected children and 8 sibling controls	Sub-analysis of children aged 2 to 12 years; Swazi Demographic and Health Survey Request for referral of HIV-infected children with HIV-uninfected mothers	Injections, infusions and “informal” surgical procedures (removal of sand fleas) were more common in cases than controls.
Vaz *et al*. [9]	22/450 (4.9%) 10/450 (2.2%) once blood transfusions excluded HIV-infected children	Retrospective review University Paediatric HIV reference Centre, Maputo, Mozambique	12 likely transfusion-related
France, Frange *et al*. [20]	Five HIV-infected children with negative HIV PCR tests until 6 months of age	Retrospective analysis of 4539 children	Premastication and breast-feeding excluded. Three cases diagnosed between 14 and 18 months, two at 10 and 12 years. Phylogenetic strain analysis in the 12-year-old showed homology with maternal virus. Risk factors included poor socio-economic backgrounds and sustained HIV-1 viremia in the mothers during the follow-up.
USA, Gaur *et al*. [7]	Three HIV-infected children	Case series, 2 from Miami, FL, and 1 from Memphis, TN.	First report identifying premastication of food as a route of transmission
South Africa, Slogrove *et al*.	10 HIV-infected children	Second case series from Tygerberg Children's Hospital, Western Cape	Eight hospitalized, 3 as neonates. Premastication in 1 with HIV-infected parents. Surrogate breastfeeding in 3. Breast milk donors not tested.

## Discussion

### Our own experience

We first became aware of this problem in 1998 when asked to investigate three children with HIV-uninfected parents at a regional hospital near Cape Town, South Africa. We confirmed the HIV-negative status of parents and siblings in all three families. However, subsequently we documented HIV infection in a previously uninfected sibling. The likely explanation was that the HIV-infected index patient developed epistaxis together with chronic otorrhea when the younger brother had herpes stomatitis. The siblings slept in the same bed. Relatedness of HIV was confirmed by phylogenetic linkage studies [10]. We were unable to identify obvious routes of transmission for the three index children. In 2000, we established a registry to document additional cases [11]. In an audit of HIV-infected children from our clinic between 1997 and 2001, we noted that 8 (2.9%) of 274 children had HIV-uninfected parents [12].

We reported 14 cases from the registry in 2004 [13]. All had HIV-uninfected parents. Two children came from other provinces and two were from Red Cross Children's Hospital, also in Cape Town and previously reported [14]. Thirteen children had a history of previous hospitalization, 10 during the first month of life. We postulated that poor infection control could be implicated as we had observed many deficiencies in this area. These included using multi-use heparinised saline bags for flushing intravenous lines and the re-use of blades for shaving the scalp prior to inserting intravenous cannulae. Poor handling of expressed breast milk for premature infants was also noted. Expressed breast milk had been poorly labelled and breast pumps were shared without proper disinfection between users. This phenomenon was well documented in a survey of milk rooms in the Free State Province of South Africa [15].

Since 2004, we reported an additional 10 children with probable non-vertical, non-sexual HIV infection [16]. Nine had HIV-uninfected parents. All children had been previously hospitalized in seven hospitals. Two had confirmed episodes of surrogate breast-feeding, but with the status of the “donors” unknown. Sexual abuse was excluded by interview and examination in three children and only by interview in seven children. Two children had received blood transfusions from confirmed HIV-seronegative donors. Poor infection control practices in hospitals were again implicated as the likely route of transmission. One child had HIV-infected parents and was not breast-fed. The father, non-adherent to anti-retrovirals, had given the child pre-masticated food during the weaning period. The mother denied surrogate breast-feeding and history of sexual abuse was excluded.

### Experience in other resource-limited settings

There is conflicting evidence about the scale of this problem. For example, Biraro *et al*. reported an epidemiological study of 6991 children and their mothers in rural southwest Uganda. The prevalence of HIV in children above 18 months of age, when antibody detection is reliable for infection status, was 0.37% (26 of 6981). Depending on the level of certainty, between one and three children had non-vertical, non-sexual transmission. Therapeutic injections, including blood transfusions were more likely in children probably infected through MTCT, suggesting that these children were sicker rather than a potential for exposure to contaminated blood or equipment [17]. Similarly, in national survey data from Uganda (2004–2005), seven (0.1%) children below 5 years of age of 8355 HIV-uninfected mothers were HIV-infected, supporting a low but real risk of non-vertical, non-sexual transmission in Uganda [18]. Vaz *et al*. described 22 HIV-infected children with HIV-uninfected parents, comprising 4.8% of their clinic cohort in Maputo, Mozambique. They identified blood transfusions as a possible risk factor in 12 of the 22 children [9].

The extent of non-vertical, non-sexual infections could be far higher in some areas. For example, in an analysis of the 2006–2007 Swaziland Demographic and Health Survey, 11 out of 50 HIV-infected children had HIV-seronegative mothers. The same authors also identified eight children in Kenya with HIV-seronegative mothers through seeking referrals from doctors and nurses [19].

### Experience in well-resourced settings

In France, Frange *et al*. [20] described late HIV infection in 5 of 4539 HIV-exposed children born to HIV-infected women. Polymerase chain reaction (PCR) testing showed that all were HIV-uninfected until 6 months of age in the absence of breast-feeding. In the Unites States, the Centres for Disease Control and Prevention (CDC) reported that in 2009, 35 (21%) out of 166 paediatric HIV infections below 13 years of age were not maternal. Transmission categories were listed either as haemophilia, blood transfusion or “risk not reported or identified” [21]. Transfusion-related transmission seems unlikely, but cases could also reflect inadequate data collection to exclude sexual transmission.

The first report implicating premastication as a potential risk factor for HIV transmission [7] was followed by a survey from the Western Cape, South Africa and United States showing that premastication is widely practiced in some communities [22,23].

### Health-care associated transmissions and injection safety

Inadequate knowledge of blood-borne virus transmission risk seems prevalent among health care workers and the general population. Infection control practices are often poorly implemented and remain undervalued in many HIV-endemic settings. In particular, unsafe injection practices are a major problem. In 2000, Hutin *et al*. [24] estimated that 16 billion medical injections were administered worldwide, of which 6.7 billion (39%) were given with re-used equipment. This potentially translated into 21 million hepatitis B virus infections (32% of new infections), 2 million hepatitis C virus infections (40% of new infections) and 260,000 HIV infections (5% of new infections) per year [25].

Surprisingly, there are numerous reports of blood-borne virus transmission in hospital settings through poor infection control in resource-rich settings. Re-use of syringes [26], multi-use vials [27–29] or saline bags [30] and inadequate sterilisation of instruments [31,32] are implicated. Even in the United States, unsafe injection practices still occur on a large scale. Pugliese *et al*. reported that 1.1% of 5446 respondents re-used a syringe to enter a multi-dose vial and then saved that vial for another patient [33]. The data from Africa, although sparse, is nonetheless equally concerning. Multi-use of syringes after changing needles has been documented in the setting of inadequate supply of syringes in Swaziland [34], and 44% of health care workers in northern Cameroon reported re-using syringes [35]. Pépin *et al*. recently reported an outbreak of hepatitis C virus and human T cell lymphotropic virus-1 (HTLV-1) in Equatorial Africa linked to chemoprophylaxis administered by intramuscular injection for trypanosomiasis prior to 1951. They also hypothesize that widespread unsafe injection practices could have facilitated the initial spread of HIV-1 [36]. The same researchers, in a cross-sectional study of 1608 individuals over 55 years of age, had previously linked multiple injections for tuberculosis or trypanosomiasis to HIV-2 infection (for women, cliterodectomy was an independent risk factor) [37,38]. This experience is very similar to that reported by Strickland of hepatitis C infection following mass injection for trypanosomiasis in Equatorial Africa [38].

As an extreme example, large iatrogenic outbreaks of HIV have been associated with non-sterile medical equipment usage in children from Kazakhstan and Uzbekistan [39,40]. In a similar nosocomial outbreak in Libya, 37 children were HIV infected. One-third had serological evidence of hepatitis B and 47% had hepatitis C [41].

The WHO has developed a toolkit for assessing injection safety [42]. The tool is mainly reliant on observation of procedures rather than eliciting information about unsafe practice in sensitive key informant interviews or from anonymous surveys. Therefore, it is likely to be biased and may not accurately document unsafe practices, as has been reported in Swaziland and Cameroon [34,35]. Other measures implemented to improve injection safety include re-use prevention devices such as auto-disable syringes for immunization programs since 1999 [25]. Donors responsible for vaccine procurement should also ensure safe injection practices.

### Intra-household transmission of HIV

Apart from our own case report [10], intra-household transmission is well documented. French *et al*., from Australia, reported probable spread between two sisters who shared a bathroom and sometimes also the same razor to shave body hair and from a male with psoriasis to his mother, who provided topical care without gloves [43]. In a review of demographic data from Mozambique, Brewer concluded that traditionally circumcised and scarified children and youth were two to three times more likely to be infected with HIV than those without these risk factors [44].

## Conclusions

There appears to be a low but real risk of non-vertical, non-sexual HIV transmission (and other blood born viruses) to children. The need to decrease risks associated with hospitalization and medical care is well recognized [45], but very few initiatives have addressed this problem in resource-poor settings. Guidelines for the management of expressed breast milk have already been developed and should be widely disseminated [46] but more effort should be invested to disseminate best practice guidelines for preventing blood-borne virus transmission, especially in settings with high disease burdens. Studies to document current practice, raise awareness of high-risk practices and identify pragmatic interventions to reduce transmission risks should be prioritized.

## Abbreviations

HIV, Human immunodeficiency virus; MTCT, Mother to child transmission; CDC, Centres for Disease Control and Prevention; USA, United States of America; WHO, World Health Organization.
